# Identifying economics’ place amongst academic disciplines: a science or a social science?

**DOI:** 10.1007/s11192-017-2519-3

**Published:** 2017-09-13

**Authors:** John Hudson

**Affiliations:** Department of Economics, University of Bath, Bath, BA2 7AY UK

**Keywords:** Economics, Journal titles, REF, Cluster analysis

## Abstract

Different academic disciplines exhibit different styles, including styles in journal titles. Using data from the 2014 Research Excellence Framework (REF) in the UK we are able to identify the stylistic trends of different disciplines using 155,552 journal titles across all disciplines. Cluster analysis is then used to group the different disciplines together. The resulting identification fits the social sciences, the sciences and the arts and humanities reasonably well. Economics overall, fits best with philosophy, but the linkage is weak. When we divided economics into papers published in theory, econometrics and the remaining journals, the first two link with mathematics and computer science, particularly econometrics, and thence the sciences. The rest of economics then links with business and thence the social sciences.

## Introduction

Is economics a science or a social science? Arguments have been made for both orientations, i.e. that economics is a social science (Frey 1999), a science (Frey 1999) and even a moral science (Schabas 2009). In terms of sciences it has been linked with physics, with many physicists doubling as, or transforming into, economists and a subpart of physics, econophysics, specifically evolving which deals with economics (Stanley et al. 1999). There are also links with biology (Marshall 1920; Daly 1968). Frey (1999) argues economics is a social science as it is part of those sciences which deal with actual problems of society, i.e. it is the subject matter which makes it a social science. However, he also points out that in practice economists tend to fill the journals with axioms, lemmas and proofs, i.e. they adopt what they perceive as a scientific, and particularly a mathematical, methodology. Mayer (1980) argues that economists act as though economics is a hard science and this is reflected by their sophisticated use of mathematics. However, he then goes on to argue that econometrics is not sufficiently advanced to enable us to test theories as a hard scientist would. With respect to this argument, it is worth noting that econometrics has moved a long way since 1980, when Mayer wrote his paper and perhaps that objection is not as valid today as it was then. In any case, part of the reason for this disagreement as to the nature of economics may be because the subject matter of economics deals with people, whether as agents in firms or consumers, either as individuals or aggregates. This will tend to place it as a social science. But in terms of methodology it has arguably closer links with the sciences. It is essentially a quantitative discipline. Theoretical models are built to replicate and simplify some real world situation. Empirical work is focused on econometrics and many of the techniques used and developed by economists are also used outside the discipline and often in the sciences, such as biological sciences.

Economics has of course evolved over the years. Fourcade et al. (2015) argues that for much of the period since 1945, a theoretical approach based on rigorous mathematical modelling was the main path to establishing scientific purity in economics. However, in the 1990s and 2000s there occurred a shift in emphasis from theory to empirical work. Einav and Levin (2014) potentially link this shift to a substantial increase in data availability. They argue that this may change the emphasis on setting up and testing hypotheses to one of searching for the best possible explanation. It may also further move economics away from the emphasis on theory, all of which may in turn impact on how economists perceive themselves and the relationship of economics with other disciplines. The quantitative nature of most economics research is in contrast to the qualitative methods that characterize the work of many other social scientists. Mankiw (2006) epitomises this perception, when he admitted he thought of himself as, and sought to project the image of, a scientist. He admitted that this might amuse physics academics, but he would remind them that economists formulate theories with mathematical precision, collect huge data sets^1^ on individual and aggregate behaviour, and exploit the most sophisticated statistical techniques to reach empirical judgments that are, potentially at least, free of bias and ideology. However he also thought that economists could be likened to engineers, having been put on earth not to propose and test elegant theories, but to solve practical problems. This perception that economics is different from the social sciences may also have contributed to the insularity of the discipline, as reflected for example in the relative dislike of economists for interdisciplinary work when contrasted with other social scientists (Fourcade et al. 2015), as well as a reluctance to cite work from these other disciplines (Romer 2015; Pieters and Baumgartner 2002).

There have been a number of attempts to map academic disciplines on the basis of citation or similar data. It is often done for specific disciplines, rather than science as a whole. Thus McCain (1991) analyses co-citation data,^2^ using cluster analysis and multidimensional scaling, to produce maps of highly cited and co-cited documents in clusters representing interrelated research activities. The focus is on mapping economics journals, and is able to identify several major groups comprising: the ‘core of economics’, a West European set of journals, formal journals, basically key theory and econometrics journals, public finance journals and economics journals.^3^ A number of studies do attempt to map the full range of disciplines. Rafols et al. (2010) using a matrix of similarity measures based on correlations of different pieces of information items, conclude that science is a fragmentary structure composed of both solid clusters and empty spaces. This gives support to the concept of independent, but potentially linked disciplines, rather than a continuum of study with somewhat artificial boundaries. The analysis is based on citing similarities among ISI subject data. There is an important biomedical research cluster and a major physical sciences cluster, comprising engineering, physics and material sciences. There is a rather diffuse social sciences cluster, which Rafols et al. argue is due to relatively low citation rates. Economics is part of this cluster, but with some links to mathematics and computer science. Bollen et al. (2009) use click stream data to construct a journal network that outlines the relationships among various scientific domains. Economics is on the periphery of the social sciences cluster. But it is also one side of a bridge connecting the social sciences to physics and engineering via production and manufacturing.

Hence these mappings tend to loosely tie economics to the social sciences, but often with some linkage to parts of the sciences. However most of these analyses are based on citations or similar measures, rather than the similarities in the structure of academic work. We have already emphasised that economics analyses social science issues, but that it is the methodology or the style of working which differentiates it from the social sciences and links it to the sciences. It is almost inevitable that economics’ citations will be greater in social sciences, than the sciences. But this tells us relatively little about the similarities between styles of working. There is little hard evidence on subject linkages based on different styles and in order to provide the beginnings of a perspective on this, we compare the journal title characteristics of the different disciplines. The characteristics we will focus on include title length, average word length, the proportion of papers containing a colon, the proportion containing a question mark, the average publication date and the number of authors. In particular we will extract the economics’ footprint as reflected in these title characteristics. We will then compare this footprint with those of other disciplines. We will argue that each discipline tends to adopt a certain style in terms of these characteristics. Lewison and Hartley (2005) argue, for example, that there are substantial differences in title length, use of colons and question marks, etc. between disciplines. Nagano (2015) also emphasises different disciplinary conventions with respect to the title, distinguishing between the hard (e.g. medicine and engineering) and soft (e.g. the social sciences, including economics) sciences. Different disciplines also tend to differ in the number of authors and the extent to which journal papers are important compared to, for example, books. If disciplines have their own style, then it seems reasonable to assume that there are also similarities between contiguous disciplines. For example, we would expect, as suggested in the literature, the characteristics of a social science discipline to be more closely aligned with other social science disciplines than ones in the sciences. Following on from this, we will then use cluster analysis to place the different disciplines within groups to see how well they fit into the social science, science and art and humanities categories. In the process we will also identify the group and subjects to which economics most closely belongs.

In doing this analysis we make use of a unique data base obtained from the Research Excellence Framework^4^ (REF) of 2014. This is the latest in a series of exercises seeking to evaluate the quality of research done in UK universities across a range of subject areas – termed units of assessment (UoAs). In this REF there were 36 such UoAs, as listed in Table 1, including one for economics and econometrics.^5^ Table 1 also gives the number of papers on which our analysis is based. In economics there were 2600 individual pieces of work submitted. Not all were journal papers and a proportion were submitted more than one once to the economics panel, i.e. by definition they were jointly authored papers. This left 2246 separate papers to analyse. Each entry to a UoA was scrutinised by a sub-panel working within the framework of 4 main panels—A–D. Economics was in Panel C along with the other social sciences. The main scrutiny was on the quality of academic research contributions, primarily journal articles and books, which were individually looked at and assessed by panel members. Each participant (faculty member) in the REF could submit up to four research outputs (e.g. papers). Each submission was also accompanied by a research environment statement and a number of case studies which highlighted the impact of the university’s research on society or the economy, widely defined (Khazragui and Hudson 2015). The evaluation thus comprised three components: (1) the quality of the four research outputs (e.g., papers) submitted by individuals, (2) the research environment statement and (3) the impact of the research on society or the economy. Our focus is on the publications. Hence, and this is an important prerequisite for the study, the definition of the discipline of the sciences and social sciences is based on the REF’s subject-classification system. In the next section we present the summary data on title characteristics. We then use cluster analysis to group the different disciplines together and finally conclude the paper.

**Table 1:** Characteristics of journal paper titles in the 2014 UK REF

	Length	Authors	Date	Colon (%)	? (%)	Papers (%)	Word length	% in UoA	% out UoA	Number of papers
Clinical Medicine	109	9	2010.47	19.49	1.107	99.89	7.64	8.92	14.98	12,193
Public Health	112	7	2010.58	58.12	6.278	99.64	7.33	9.64	20.64	4396
Allied Health	106	5	2010.58	32.03	4.073	99.03	7.5	3.81	15.81	9870
Psychology	94	4	2010.52	36.22	5.675	99.54	7.53	9.07	10.27	8264
Biological Sciences	97	6	2010.45	11.48	1.309	99.67	7.6	5.63	17.07	8098
Agriculture	107	6	2010.56	18.64	2.319	99.21	7.4	3.55	13.32	3751
Earth Systems	94	5	2010.36	26.53	3.749	99.02	7.33	6.65	10.88	4855
Chemistry	95	5	2010.4	22.7	1.198	99.82	8.09	5.69	11.54	4423
Physics	77	6	2010.2	14.73	1.522	99.05	7.36	10.53	6.48	5718
Maths	67	2	2010.63	9.35	0.685	96.14	7.57	4.6	3.87	6426
Computer Science	71	3	2010.59	18.4	1.015	78.55	7.78	5.19	6.15	5717
Aeronautical Eng	89	4	2010.47	13.39	0.812	99.29	7.56	1.24	10.18	4063
Electrical Eng	86	4	2010.44	10.25	0.51	99.17	7.81	1.78	10.87	3921
Civil Eng	85	3	2010.58	19.31	2.179	97.65	7.42	1.55	11.69	1331
General Eng	87	4	2010.48	13.82	0.693	98.64	7.63	2.34	10.38	8364
Architecture	85	2	2010.85	46.64	7.72	77.1	7.27	4.55	6.21	2811
Geography	91	3	2010.81	47.33	7.46	81.93	7.25	5.48	9.5	4705
Economics	64	2	2010.72	30.41	9.35	91.79	7.3	6.38	8.05	2246
Business	81	2	2010.76	52.69	11.716	95.09	7.38	12.33	4.01	10,234
Law	77	1	2010.78	54.6	18.877	62.26	6.89	1.68	1.45	3401
Politics	78	1	2010.85	60.03	19.135	70.1	7.12	2.53	2.37	3005
Social Work	89	2	2010.81	65.53	15.987	76.91	7.13	2.67	8.13	3603
Sociology	81	1	2010.77	66.28	12.639	75.93	7.1	1.25	8.39	1978
Anthropology	84	2	2010.76	60.76	11.69	67.12	7.18	0.89	6.13	1343
Education	90	2	2010.84	61.04	13.211	77.69	7.33	4.14	4.46	4148
Sport Science	95	4	2010.83	34.93	6.475	96.57	7.34	6.22	6.71	2502
Area Studies	82	1	2010.74	63.96	12.32	56.56	7	0.2	4	974
Modern Languages	79	1	2010.84	60.12	7.578	48.21	7	1.38	2.67	2362
English Languages	73	1	2010.9	66.02	5.103	35.73	6.87	0.4	1.17	2469
History	84	1	2010.81	62.54	8.009	44.01	6.86	0.67	1.09	2822
Classics	66	1	2010.92	49.75	6.716	29	6.72	0	1	402
Philosophy	46	1	2010.96	23.31	9.07	61.67	7.25	0.74	0.45	1334
Theology	75	1	2010.78	57.37	10.225	37.06	6.82	0.17	1.21	577
Arts	73	1	2010.86	55.6	4.513	26.39	7	1.31	3.56	1662
Music	75	1	2010.95	65.18	6.614	29.63	6.93	0.87	1.74	1255
Media Studies	79	1	2010.81	65.72	9.732	52.39	7	1.35	4.05	1829
Economics sub-groups										
Theory	51	2	2010.76	16.34	4.28	99.61	7.75	6.55	5.09	257
Econometrics	69	2	2010.72	14.8	3.587	100	7.64	7.85	5.37	223
The remainder	66	2	2010.72	34.43	10.815	89.83	7.22	6.16	8.82	1766

Columns (1) median character length of title, (2) median author numbers, (3) average publication date, (4) % using a colon, (5) % using question mark, (6) % of submissions which are journal papers, (7) median word length, (8) % of multiple publications in same UoA, (9) % of multiple publications in other UoAs, (10) number of journal papers in UoA which form the sample for our analysis. Source: calculated from data on journal papers in the REF

### The differing characteristics of disciplines in terms of journal titles

Table 1 shows the characteristics of the titles, i.e. median title length, median word length, proportion of papers containing a colon or a question mark, the average date of submitted papers, etc. These calculations are done for the main subject groupings. The data relate to journal titles only and these form the basis for the cluster analysis.^6^ Focusing on journal papers, the penultimate two columns show that in economics 8.05% of papers were also submitted in other disciplines, whilst 6.38% of papers were submitted at least twice within the economics UoA. In the latter case they would most likely have been submitted by co-authors in different institutions. Multiple submissions of the same paper across disciplines were particularly common in the health and engineering UoAs. They were much less common in the arts and humanities. Multiple submissions within a discipline were relatively common in the sciences, particularly physics, and also business, but less common in engineering UoAs. Apart from the data on multiple submissions, the other columns are calculated on papers rather than submissions. Hence if a paper was submitted more than once to the economics panel, it would still only be counted once in calculating the statistics for these columns. The number of papers this left for analysis is shown in the final column of the Table. The median number of authors in journal articles for economics was 2, this is on the low side with a median across all disciplines of 3. The average was much higher at 11, being pushed up by the very high average number in physics, 92.4. But in general across the sciences and health disciplines the number of authors tends to be high. The average can be heavily influenced by one or two outliers. In physics for example, the most number of authors is 3269, for a paper on the Higgs boson. In the REF, this paper was submitted 13 times. This is why in this current analysis for the number of authors and also title length, average word length and the number of citations we work with the median. That is we calculate, for example, the median title length of all the titles in the sample and use that instead of the mean title length.

The average date of submission also shows systematic variations across the disciplines. It tends to be earliest for the sciences and engineering and latest for the arts. Economics is quite late. This may reflect the ease of writing a new paper in the different disciplines. In the sciences a grant is often needed and also the team of people tends to be larger than in the non-sciences. Thus in the non-sciences it may be easier to write a new paper if current REF output is considered unsatisfactory. However in order for the ease of writing new papers to explain the timing of submissions, we also need the starting date for research to be influenced by the REF cycle (Hudson 2016).

The other columns provide us with information on the way economists work in comparison with other disciplines. Firstly the proportion of submissions which are journal papers was quite high at 91.79%. This reflects the importance economists place on journal papers. Other submissions include books and also working papers. However, the proportion of journal papers is still higher across much of the sciences and engineering. But it does tend to fall substantially for other disciplines such as law, history and languages. Thus in classics, less than a third of submissions are journal papers.

There is also information which helps us compare economics with other disciplines in the structure of the title. Economists tend to be parsimonious in title length relative to other disciplines, with a median number of characters of 64, only philosophy having a shorter median title length.^7^ The longest titles tend to be in the health disciplines and sports sciences, which is linked to health. Economists are however less parsimonious in their use of colons. The heaviest users of colons tend to be the arts and social sciences. In sociology over 66% of submissions feature a colon and in business it was almost 53%. Colons were least common in mathematics and electrical engineering. The same is true with respect to question marks. Finally economists tend to use shorter words than the science and health disciplines and longer ones than the arts and social sciences.^8^

Hence we can see that different disciplines have different styles and practices with respect to journal titles, together with the number of authors. The sciences, including the health sciences, tend to be characterised, for example, by a large number of authors and be parsimonious in using question marks and colons, even though their titles tend to be relatively lengthy. This raises the possibility of categorising economics with respect to other disciplines, at least with extent to title characteristics, the number of authors and the submission dates for the REF, by the use of cluster analysis. The basic assumption is that people in contiguous disciplines work in a certain style which tends to characterise those disciplines. Two disciplines which are close will tend to have similar characteristics in terms of numbers of authors and the characteristics of the title. Apart from providing information on different title characteristics across disciplines, it is also worth noting that characteristics such as title length, use of colons and question marks have been found to impact on citations (Haslam et al. 2008; Jamali and Nikzad 2011; Van Wesel et al. 2014; Hudson 2016).

### Grouping disciplines

**Fig. 1:**
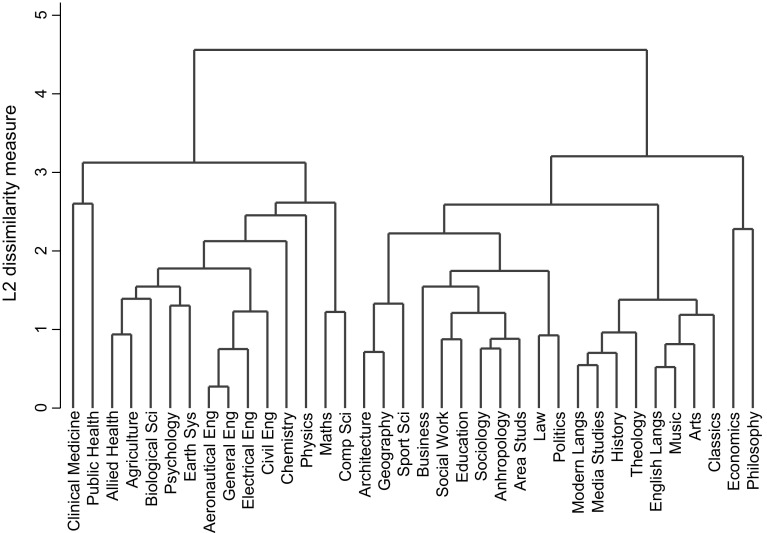
Dendrogram based on average linkage

We use cluster analysis to group disciplines based on title length, number of authors, date of submission, use of colons and question marks, word length of journal titles and proportion of submissions in the form of journal papers. The basic idea of cluster analysis is to group, first, the two disciplines that are closest using a Euclidean distance criterion. In some techniques the squared Euclidean distance is used. The Euclidean distance is formed by finding the difference in the values of the first variable (e.g., title length) between two disciplines and squaring the result, repeating for each variable, adding the all the squared differences together and taking the square root of this. Out of all the disciplines in the study, the pair that are closest join first and lowest in the dendrogram (see Fig. 1). When groups are being joined together, some measure of the collection of values of a variable is needed and various possibilities are discussed later.

We consider only journal articles, and not other publications such as books which may in themselves follow a different format in their title than do journal papers. The percentages of joint papers shown in the penultimate two columns of Table 1 were not included in the cluster analysis as they will tend to reflect the number of authors, and hence this would be to an extent double counting this characteristic. In addition, the number submitted outside the discipline (the penultimate column of the table) partly depend upon how the boundaries are drawn by the REF itself, rather than inherent characteristics of the discipline. We standardised the data by transforming each variable into a Z variable. This is frequently done when the different variables are substantially different (Everitt et al. 2011). This was done in STATA and the algorithms used to calculate the different clusters using different assumptions were also those which are given in STATA. Figure 1 shows the dendrogram calculated using the method of average linkage. As its name suggests, this uses the average over all papers in a cluster for each variable as the measure of cluster location. The average linkage method tends to work well in many situations and is reasonably robust (Kaufman and Rousseeuw 1990). The diagram shows the Euclidean distance. Two subject matter areas that have highly similar characteristics join low in the diagram, hence aeronautical and general engineering are highly similar. At higher values of the vertical axis, clustered pairs are joined by a third area, or group of areas, with largely similar characteristics to the pair. Other areas that have somewhat similar characteristics are also paired. Moving up the diagram, the number of clusters reduces and each contains more heterogeneous, although somewhat similar, areas.

Economics is initially grouped with philosophy, although quite late in the clustering process, hence the linkage is not that strong. At the same approximate level of dissimilarity is a second group involving architecture, geography, sports science, business, social work, education, sociology, anthropology, area studies, law and politics. These correspond closely to the social science disciplines and REF panel C. This group is subsequently linked to a second grouping comprising modern languages, media studies, history, theology, English language, music, the arts and classics, which largely correspond to the arts and humanities and REF panel D. It is these two combined groups that economics and philosophy eventually are linked. There is then one further large grouping. This is made up of sub groupings of allied health, agriculture, biological sciences, psychology and earth systems, which largely come from Panel A, the health sciences, and the four engineering disciplines which come from panel B, the sciences. These two sub groups merge and are then joined by chemistry, physics, a sub group of maths and computer science, and finally clinical medicine and public health. To a large extent the clusterings are intuitively plausible. But there are some surprises. For example, the clustering of sports science with architecture and geography. However this does take place quite late which suggests that the linkage with sports sciences and the other two disciplines is not that strong. Even so there are plausible connections, with geography forming a bridge between the other two. The full title of sports science is sport and exercise sciences, leisure and tourism, and the latter two areas make the linkage with geography plausible. Similarly with geography and the last two elements of architecture, built environment and planning. But the linking of psychology, psychiatry and neuroscience with earth systems and environmental sciences is more difficult to rationalise. However the subsequent linking of this subgroup with allied health, biological sciences and agriculture, veterinary and food science is easier to rationalise, e.g. the link of earth systems with agriculture and biological sciences.

Hence we can see that groupings based on journal paper characteristics categorise the different disciplines into the sciences, social sciences and the arts in a manner which largely supports the conventional groupings, as can be seen from Table 2, where these clusters are listed. We choose seven clusters^9^ as this allows the main clusters to emerge, given that some UoAs such as clinical medicine will tend to be on their own, not linking to other UoAs. In this case both the social sciences and the arts and humanities are well identified. Most of the sciences in both panels A and B form a single cluster, with the exceptions being clinical medicine, public health, chemistry and physics. Mathematics and computer science also form their own sub-group. Economics does not fit in well with the other social sciences, nor indeed does it fit in with the sciences. It fits best with philosophy, although the fit is not that strong and if we move to nine clusters it is on its own, not linked with philosophy. If we use other methods^10^ to identify the clusters, the results viz a viz economics change relatively little as can also be seen from Table 2. For example, when using Ward’s method, there is a clearer split identifying panels A and B, and the social sciences form two clusters—which join together again when we reduce the number of clusters to 6. The dendrograms give more insight than the simple identification of clusters, but what this Table also reveals is that while the contents of a cluster might be identical or almost identical under different linkage assumptions, the ordering of clusters depends on the algorithm and other assumptions. The final column shows the clusters using average linkage when we omit the number of authors. The results are similar to before, although social sciences now has two sub clusters and in panel B, chemistry and physics are now part of the other sciences, but with maths and computing excluded. But still the clusters are based around the different REF panels and hence these results are not being driven by large differences in the number of authors between disciplines. The same is true if we omit the proportion of papers which are journal papers.^11^

**Table 2:** The panels, and UoAs in the 2014 UK REF

Unit of assessment (UoA)	Clusters				
	*A*	*B*	*C*	*D*	E
Panel A Health Sciences					
1 Clinical Medicine	1	1	5	4	1
2 Public Health, Health Services & Primary Care	2	1	2	4	4
3 Allied Health Professions, Dentistry, Nursing & Pharmacy	3	1	6	4	1
4 Psychology, Psychiatry & Neuroscience	3	1	6	5	1
5 Biological Sciences	3	1	6	5	1
6 Agriculture, Veterinary & Food Science	3	1	6	4	1
Panel B Sciences					
7 Earth Systems & Environmental Sciences	3	1	6	5	1
8 Chemistry	3	2	6	5	1
9 Physics	3	2	4	5	2
12 Aeronautical, Mechanical, Chemical & Manufacturing Engineering	3	2	6	5	1
13 Electrical & Electronic Engineering, Metallurgy & Materials	3	2	6	5	1
14 Civil & Construction Engineering	3	2	6	5	1
15 General Engineering	3	2	6	5	1
10 Mathematical Sciences	4	3	6	6	3
11 Computer Science & Informatics	4	3	6	6	3
Panel C Social Sciences					
16 Architecture, Built Environment & Planning	5	5	7	7	5
17 Geography, Environmental Studies & Archaeology	5	4	7	7	5
19 Business & Management Studies	5	4	7	7	5
20 Law	5	4	7	2	5
21 Politics & International Studies	5	4	7	2	5
22 Social Work & Social Policy	5	4	7	2	5
23 Sociology	5	4	7	2	5
24 Anthropology & Development Studies	5	4	7	2	5
25 Education	5	4	7	2	5
26 Sport & Exercise Sciences, Leisure & Tourism	5	5	7	7	5
18 Economics & Econometrics	7	6	3	1	7
Panel D Arts & Humanities					
27 Area Studies	5	4	7	2	5
28 Modern Languages & Linguistics	6	7	7	3	6
29 English Language & Literature	6	7	7	3	6
30 History	6	7	7	3	6
31 Classics	6	7	7	3	6
33 Theology & Religious Studies	6	7	7	3	6
34 Art and Design: History, Practice & Theory	6	7	7	3	6
35 Music, Drama, Dance & Performing Arts	6	7	7	3	6
36 Communication, Cultural & Media Studies, Library & Information Management	6	7	7	3	6
32 Philosophy	7	6	1	1	7

Cluster A fitted by average linkage; B, ward’s linkage; C, single linkage; D complete linkage. E is fitted by average linkage excluding the number of authors

### Splitting up economics

In this section we divide the economics papers into three groups, those published in theory journals, those published in econometrics journals and all the remaining papers. We used the same categorisation principle as Hudson (2013), i.e. a journal was counted as a theory journal if some version of the word ‘theory’ appeared in the title and similarly for econometrics with respect to variations on ‘econometrics’. For this exercise, the journal *Econometric Theory* was counted as an econometrics journal. Table 1 shows the summary data on these three sub groups of economics. There are substantial differences, with titles of both theory and econometrics journals having significantly, at the 1% level, lower proportions of papers with both colons and question marks. The titles of papers in theory journals are exceptionally parsimonious, whilst their average word length is high relative to other disciplines. Although not as high as papers in theory journals, word length in econometrics journals also tends to be high relative to other disciplines. The penultimate column shows that a submission which spans disciplines is relatively uncommon for papers in theory and econometrics journals, but slightly more common in the rest of economics.

**Fig. 2:**
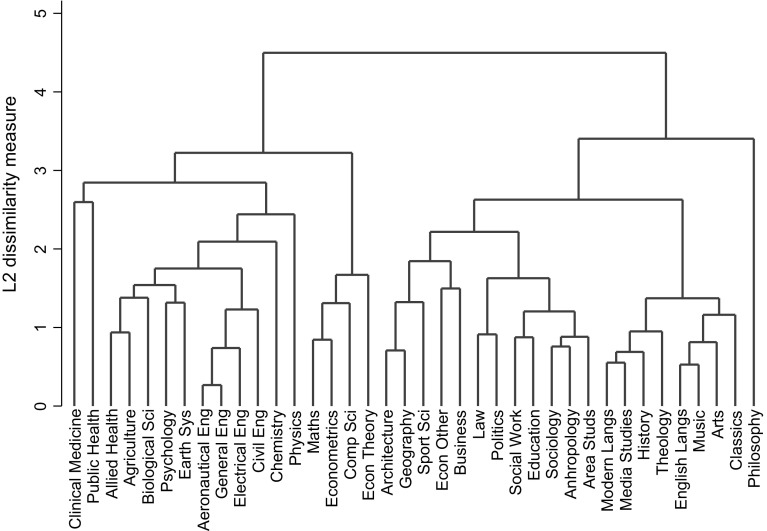
Dendrogram based on average linkage with sub-groups for economics

Redoing the cluster analysis in a manner consistent with that shown in Fig. 1 results in the dendrogram shown in Fig. 2.^12^ Papers published in econometrics journals are linked in with maths and computing as subsequently are papers published in theory journals, they thence join with the sciences, although at a high level of dissimilarity. The rest of economics now links with business and thence to the social sciences. This result does not change if we use the other methods for clustering. The clustering algorithm is a hierarchical one and expanding the disciplines has had only limited repercussions on the structure of the other clusters, and this involves mainly business and philosophy. The close tie of econometrics to mathematics is not that surprising, although that with computer science a little more so. Perhaps the most surprising conclusion is that “other economics” ties with business and follows a completely different path up the dendrogram to that of theory and econometrics. It is this second part which is linked with the social sciences. These differences suggest that economics is a broad discipline compared with some others, although it is also a discipline with a degree of heterogeneity. It is also not surprising that these clusters are different to those that we get using citation analysis (Rafols et al. 2010). The latter will relate primarily to subject matter rather than to academic style of working.

The REF data provides more information on the linkages between economics and other subjects via the submission to other UoAs of papers also submitted to the economics UoA. The proportion of joint submissions, shown in the penultimate column of Table 1, for the theory and econometrics journals combined is significantly lower, at the 1% level, than for the rest of economics.^13^ Ignoring Business UoA ‘partners’, for theory and econometrics seven out of twelve ‘partners’ were in mathematics or computer science. For the rest of economics the spread was wider. Out of 71 non-business UoA ‘partners’, nine were in the mathematics or computing UoAs, with the other largest groupings being public health (11), history (10), social work and social policy (8) and education (7). These linkages will partially reflect closeness of subject matter, as in citations, but also possibly closeness in working styles.^14^ The results, albeit with small samples, again suggest that the more quantitative part of economics is closely linked with mathematics and computing, but that the rest of the discipline is spread more widely across parts of the sciences, the social sciences and also the arts.

## Conclusions

This paper represents one of the first attempts to identify, using quantitative analysis, the linkages between different disciplines based on the stylistic characteristics of journal papers. We are able to identify subjects, as defined in the REF, into clusters which closely resemble those which form the four main REF panels and also more generally what are thought of as the sciences, the social sciences and the arts and humanities. This indicates both that contiguous disciplines have similar style characteristics evident in the title of the journal papers in those disciplines, and that using these we are able to link the different disciplines together in a manner which broadly is reflective of the social sciences, sciences and arts and humanities as they are generally understood. Thus linking cluster 3 to panels A and B, cluster 5 to Panel C and cluster 6 to Panel D as in column A of Table 2, correctly identifies 29 of the 36 disciplines. The disciplines of physics and public health however, are difficult to categorise and maths and computer science form a separate group from the rest of the sciences. Economics as a whole is linked with philosophy, although not strongly so. Only area studies is allocated to a ‘wrong’ panel. The fact that we have been able to successfully allocate disciplines to clusters which so closely resemble the REF panel groupings suggests that our methodology is sound.

On the basis of our analysis, it seems possible that most of economics does not closely link with any of the other disciplines. Economics tends to have relatively few authors, but more than the arts and humanities, tends to be parsimonious in title length and to an extent, particularly when compared with the other social sciences and the arts and humanities, in the use of colons. When we look at sub-disciplines within economics, we find that econometrics first links with maths and these are then joined by economic theory and computer science. Business and the rest of economics link together and thence with the rest of the social sciences, although at a relatively high level of dissimilarity and hence the linkage is not that strong. However there is little evidence of a link with the sciences per se. This is not unexpected, large grants involving several labs have become increasingly important in the sciences, at least as represented by the leading papers (Galison and Hevly 1992). This may have tended to increase author numbers and this too will leave a footprint on title styles (Hudson 2016). Of course grants are important in economics too, but not to the same extent as in big science.

With respect to the question posed in the title of this paper, our view, in part informed by these results, is that the subject matter of economics places it clearly as a social science, but many in the discipline act as if it were a science. Economics has gradually developed into an interdisciplinary subject that uses scientific methods, for example setting out and formally testing hypotheses, and particularly a mathematical approach, to solve social science problems. This dual aspect may explain why it fails to fit neatly with either as in fact it combines elements of both. It is a social science pursued with more quantitative rigour than much of the rest of the social sciences. This emphasis on technique then pushes economics and economists away from the other social sciences and in some sub-disciplines towards maths. Placing economics as a science, emphasises technique above subject matter. But there is then a danger that technique becomes an end in its own right, rather than a means to an end, i.e. a vehicle which allows a more refined analysis of economic issues which have wider relevance. Often, for example when developing a new econometric technique which subsequently becomes widely used, this is justified. But sometimes this is not the case, which helps explain the relative isolation of the discipline as noted earlier. This does not mean that economists should abandon their quantitative focus, but that they should always strive to ensure that their work contributes, directly or indirectly, to an understanding of a real world issue. This is consistent with Frey’s (1999) observation that economics is part of those sciences which deal with actual problems of society, but that most economists attempt to imitate the sciences and that economics can be regarded as a branch of applied mathematics. It is also consistent with the view that that economics, particularly neoclassical economics, is the most mathematical of the social sciences (Porter 1996).

There are two further implications of this, both of which might be explored in further work. Firstly, modern universities have mainly organised their structure along the ‘tree of knowledge’ type model (Lenoir 1997), according to which knowledge is split into branches (for example, sciences, social sciences), then into major disciplines and thence into sub-disciplines and specialties. This has tended to result in economics being linked with the social sciences or business schools. Seldom are they linked with any of the sciences. This may lead to problems in being involved with administrators and academics who do not really understand the way economists work, nor their values. Secondly, the ethos economists perceive themselves as being part of tends to set the confines within which successful economists must work. If economists perceive themselves as mathematicians, then this sets out the theoretical style we see in many journals. If they see themselves as scientists then the emphasis moves to hypothesis testing (Mayer 1980). If, on the other hand, they see themselves as social scientists this will focus attention more on policy outcomes and a greater readiness to use the results of the other social science disciplines.

The analysis has focused on papers submitted to the REF. Whilst this provides a large number of papers to analyse, they are biased towards the better, higher quality papers. Further analysis could extend the data set to cover a more random set of papers across a range of journals in each discipline in order to determine whether the writing characteristics of these high quality papers translate to the discipline as a whole. In addition, further work could estimate clusters based on other aspects of papers, such as length and the typical structure of a paper. Abstracts too differ between disciplines. In many journals, it is a single paragraph of between 100 and 250 words. But in some of the science disciplines the abstract too is divided into sections. Other possible characteristics include the numbers of equations, footnotes or endnotes, quotations and use of explanatory diagrams or flow charts, use of regression analysis and the ratio of the length of the concluding section to the rest of the paper.^15^ Finally, we have divided the subject along into theory, econometric and other areas. This could have been taken further, dividing by subject subfields, such as health economics, labour economics and macroeconomics, and then studying these subfield’s attribution by means of semantic similarity. Defining the subfields could be done, e.g., by looking at title papers in journals recognised as representing the different disciplines.

## Footnotes

1 Of course Mankiw’s statement needs to be qualified, compared with meteorological research, for example, economists' data sets are relatively small.

2 Co-citation data relates to the number of times two ‘works’, broadly defined, are jointly cited. This can be two specific papers or journals. Alternatively, author co-citation focuses on the number of times any two authors are cited together.

3 There is potentially a degree of overlap between these clusters. Thus *Review of Economic Studies* is included in the formal journals and the *Economic Journal* in the West European ones. Intuitively both could have been categorised as part of the core.

4
http://www.ref.ac.uk/.

5 These are abbreviated titles. The full titles are given later in Table 2.

6 This is similar to Table 2 in Hudson (2016). However in that paper the data relate to all UoA entries, whilst the data presented in this paper exclude multiple submissions within a discipline, i.e. papers are counted just once, even if entered several times. This is because the purpose of this analysis is to focus on differences between disciplines whilst in the earlier paper the focus was on differences between REF entries. However, the regression analyses in that paper did exclude multiple submissions. In addition, for title length, word length and the number of authors we now use the median rather than the mean.

7 This was also the case if we compared averages instead of medians.

8 The differences with the social sciences were greater when we used the mean rather than the median.

9 Corresponding to approximately 2.6 units of Euclidean distance on the dendrogram shown in Fig. 1.

10 Ward’s method is based on increasing the overall within-cluster variance to the smallest possible degree. Single linkage is based on the minimum distance and complete linkage is based on the maximum distance.

11 If we repeat the analysis but using the raw data, not the standardised one, then there are some changes. The clusters still identify the different panels well, but now economics is weakly linked with mathematics and computer science. Again there is no evidence of a linkage with either the rest of the sciences or the social sciences.

12 For this we assume that the proportion of papers published in journals is the same as for the discipline as a whole, for all three parts of the discipline.

13 The majority of these were with the Business UoA. This may not reflect economists working with non-economists as many economists are in business schools.

14 Although it is also possible these papers involve people with differing skills.

15 For example an examination of the first issues of 2016 for several journals revealed substantial differences in the use of equations. In the social sciences they were relatively rare. Unsurprisingly, in mathematics usage was very high, on occasion over 100. In core theory (*Journal of Economic Theory*) and econometrics (*Econometrica*) journals the average was approaching that of mathematics—although the latter had much more mathematical content. The number of equations in three core mainstream economics journals (*American Economic Review*, *Quarterly Journal of Economics* and *Journal of Political Economy*) was much less, although still far in excess of the rest of most of the social sciences. Details available from author.
